# Molecular Characterization of Patatin-Like Phospholipase Domain-Containing 3 (PNPLA3) Gene in Alcoholic Liver Disease

**DOI:** 10.7759/cureus.88316

**Published:** 2025-07-19

**Authors:** Shreyas N, Siddanagouda Biradar, Gurushantappa S Kadakol, Anuja Kadagud, Abhijit Hanamantaraya Utnal, Raksha Chandraiah

**Affiliations:** 1 General Medicine, Shri B. M. Patil Medical College Hospital and Research Center, Bharatiya Lingayat Development Educational Association (BLDEA) (Deemed to be University), Vijayapura, IND; 2 Anatomy, Human Genetics Laboratory, Shri B. M. Patil Medical College Hospital and Research Center, Bharatiya Lingayat Development Educational Association (BLDEA) (Deemed to be University), Vijayapura, IND

**Keywords:** alcoholic liver disease, genetic polymorphism, india, liver cirrhosis, molecular characterization, pnpla3 gene, rs738408, rs738409

## Abstract

Background: Alcoholic liver disease (ALD) is a significant cause of liver-related morbidity and mortality worldwide. The role of genetic factors, particularly the patatin-like phospholipase domain-containing 3 (PNPLA3) gene, in the progression of ALD remains underexplored in the Indian population. This study aims to investigate the molecular characterization of the PNPLA3 gene polymorphism in individuals with ALD in India.

Materials and methods: A cross-sectional study was conducted at Bharatiya Lingayat Development Educational Association (BLDE) (Deemed to Be University), Shri B. M. Patil Medical College Hospital and Research Center, Vijayapura, from May 2023 to December 2024. The study involved 60 patients diagnosed with ALD, confirmed through clinical history, radiological findings, and biochemical parameters. Genetic analysis for PNPLA3 polymorphism was performed using polymerase chain reaction (PCR)-based molecular techniques, and DNA sequencing was carried out to detect the rs738409 and rs738408 mutations. Data analysis was performed using SPSS version 26 (IBM Corp., Armonk, NY), and descriptive statistics and independent sample t-tests were used for comparisons.

Results: Among the 60 study participants, the majority were male 58(96.7%), with a mean age of 41-50 years. The most common disease stage was cirrhosis, 42(70%). Genetic analysis revealed that 49(81.7%) of participants had no detectable mutation, while 5(8.3%) had the rs738409 polymorphism, and 6(10%) had both rs738409 and rs738408 mutations. No significant associations were found between the genetic mutations and demographic or clinical variables. However, laboratory analyses showed significantly higher aspartate aminotransferase (AST) levels and lower leukocyte and platelet counts in mutation-positive individuals compared to those without mutations.

Conclusions: The study identified the presence of PNPLA3 genetic mutations in a small subset of patients with ALD in India. Although the mutations did not show significant associations with demographic or clinical variables, they were associated with altered laboratory parameters, highlighting the potential role of PNPLA3 in the progression of ALD. Further studies are required to validate these findings and understand the genetic basis of ALD in the Indian population.

## Introduction

Alcoholic liver disease (ALD) and non-alcoholic fatty liver disease (NAFLD) are among the most prevalent causes of chronic liver disorders worldwide, with rising incidence posing serious public health challenges [1]. Globally, NAFLD affects approximately 25% of the population, making it the most common liver disease, while ALD contributes to 30%-40% of liver-related mortalities [1]. In India, recent estimates suggest the prevalence of NAFLD is between 9% and 32% in the general population, and ALD accounts for nearly 24% of all liver cirrhosis cases [2]. While alcohol has long been integrated into various social customs, its excessive and chronic consumption exerts considerable harm to public health. According to WHO data, global average alcohol consumption is approximately 6.18 liters per capita per year [3,4]. ALD includes a spectrum of progressive hepatic injuries, from simple steatosis to alcoholic hepatitis, culminating in alcoholic cirrhosis, the most advanced and irreversible stage of alcohol-induced liver damage [5,6].

India, like many developing nations, is witnessing a steady increase in alcohol consumption. Although the national average per capita consumption remains close to the global average (about 12.9 liters/year), regional and demographic disparities are notable [2,7]. For example, studies show alcohol use is more prevalent in North India, with alcohol-related dependence and complications significantly higher among men compared to women [3,8]. Alcohol is implicated in a substantial burden of disease, contributing to 4% of all deaths, 20% of hospital admissions, 60% of emergency injuries, and 20% of traumatic brain injuries in India [9]. These alarming statistics underline the necessity for continued research into the pathogenesis and progression of ALD in the Indian population.

Despite widespread consumption, not all individuals who consume excessive alcohol develop advanced liver disease. Only about 6-41% of chronic heavy drinkers -- defined as consuming more than 60-80 g/day for men and over 20 g/day for women -- develop cirrhosis [10]. This suggests that additional genetic, environmental, and metabolic factors influence susceptibility to ALD. Factors such as the pattern and type of alcohol consumed, co-existing viral infections (e.g., HIV, hepatitis C virus (HCV), hepatitis B virus (HBV)), nutritional status, obesity, and metabolic syndromes can significantly modulate disease outcomes [10]. In recent years, molecular and genetic research has begun to unravel how these external factors interplay with host genetic predispositions [2,10].

One of the most extensively studied genetic contributors to liver disease is the patatin-like phospholipase domain-containing 3 (PNPLA3) gene. PNPLA3 encodes a transmembrane protein (also known as adiponutrin) primarily expressed in hepatocytes, playing a central role in hepatic lipid metabolism [1]. A single-nucleotide polymorphism (SNP), rs738409 (C>G), resulting in the isoleucine-to-methionine substitution at position 148 (I148M), has been strongly linked to increased hepatic fat accumulation, inflammation, fibrosis, and progression to hepatocellular carcinoma (HCC) in both ALD and NAFLD [1]. Additional evidence also implicates PNPLA3 in impaired triglyceride hydrolysis and altered retinol metabolism, further amplifying liver injury in genetically susceptible individuals [9].

Although the PNPLA3 I148M variant has been extensively studied in Western populations, limited data are available on its prevalence and clinical significance in the Indian context. Given the genetic diversity and rising burden of alcohol-related liver disease in India, it becomes imperative to investigate population-specific genetic markers. This study primarily aims to determine the prevalence of PNPLA3 gene polymorphisms, specifically rs738409 (I148M) and rs738408 (S453I), in Indian patients with alcoholic liver disease (ALD). Secondarily, it seeks to explore the clinical correlation of these polymorphisms with disease severity and progression. These findings will contribute valuable insight into the genetic basis of susceptibility and progression in this high-risk group.

## Materials and methods

This was a cross-sectional study conducted at Bharatiya Lingayat Development Educational Association (BLDE) (Deemed to Be University), Shri B. M. Patil Medical College Hospital and Research Center, Vijayapura, during the period from May 2023 to December 2024. The study included patients admitted with a diagnosis of alcoholic liver disease (ALD), confirmed by clinical history and examination, radiological findings on ultrasonography, and biochemical parameters including liver function tests (aspartate aminotransferase (AST)/alanine aminotransferase (ALT)). Based on an estimated prevalence of PNPLA3 SNP in the Indian population of 3.2%, and assuming a 95% confidence level, 5% level of significance, and a 5% margin of error, the sample size was calculated using the formula n = (Z² × p × (1−p))/d², where Z = 1.96, p = 0.032, and d = 0.05. The estimated sample size for the study was 60.

Patients who were HBsAg-positive, HCV-positive, diagnosed with hepatocellular carcinoma, or with drug-induced liver injury were excluded from the study. A total of 60 eligible ALD patients were included and evaluated for PNPLA3 gene polymorphism using PCR-based molecular techniques.

DNA extraction

One ml of peripheral venous blood was collected into ethylenediaminetetraacetic acid (EDTA)-coated tubes and stored at 4°C. Genomic DNA was isolated using the salting-out method, which involves the use of phenol-chloroform, and then purified via ethanol precipitation. DNA extraction was carried out using the BIO-RAD Thermocycler100 (Bio-Rad Laboratories, Hercules, California).

Isolation of genomic DNA and quantification

From 300 µL of peripheral blood, genomic DNA was isolated using a commercially available DNA kit, and quantification was performed using the Tecan multimode reader (Infinite 200 PRO Microplate readers, Männedorf, Switzerland), a micro-volume UV spectrophotometer optimized for nucleic acid and protein quantification.

Agarose gel electrophoresis

**Figure 1:**
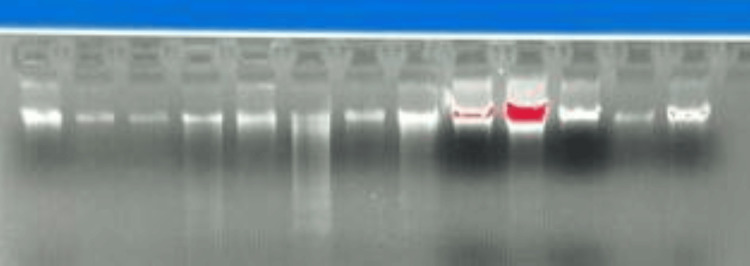
Agarose gel image of genomic DNA of PNPLA3 in ALD samples. ALD: alcoholic liver disease, PNPLA3: patatin-like phospholipase domain-containing 3.

The quality of the extracted DNA was confirmed by agarose gel electrophoresis. A 1% agarose gel was prepared by dissolving 1 g of agarose in 100 mL of 1X TAE buffer. DNA samples were also quantified using a NanoDrop spectrophotometer (Quawell, Sunnyvale, California), and both the quantity and quality were recorded (Figure 1).

Primers for exon 3 and exon 6 of the PNPLA3 gene were designed using the web-based tool PRIMER3 and validated for specificity using in silico polymerase chain reaction (PCR) software. The primers were synthesized by MWG Biotech, India (Table 1).

**Table 1:** Details of the primer sequences and annealing temperatures used for the amplification of exons 3 and 6 of the PNPLA3 gene. PNPLA3: patatin-like phospholipase domain-containing 3.

Sl. no	Primer ID	Sequence	Product size	Annealing temperature
1	PNPLA3 3F	5′-TGCTCACTTGGAGAAAGCTTATG-3′	200	56.0°C
	PNPLA3 3R	5′-CACTTCAGAGGCCCCCAG-3′		
2	PNPLA3 6F	5′-GTTTTCCGTGCCCTTCACAG-3′	252	54.5°C
	PNPLA3 6R	5′-GAGTGGGTACCTGTAGCGAG-3′		

Polymerase chain reaction (PCR)

PCR amplification was performed in a 20 µL reaction mixture containing 0.5 µL of genomic DNA (75-150 ng/µL), 0.5 µL each of forward and reverse primers (5 pmol), 0.4 µL of dNTPs (10 pmol), 0.2 µL of Taq DNA polymerase (3 units/µL), 4 µL of 5X Taq buffer (Bio-Rad Laboratories, Hercules, California), and the remaining volume was adjusted with molecular biology grade water. Amplification was conducted using an Eppendorf Mastercycler Gradient (Eppendorf, Hamburg, Germany) under the following cycling conditions: initial denaturation at 98°C for 10 seconds, followed by 35 cycles of denaturation at 98°C for 10 seconds, annealing at primer-specific temperatures for 10 seconds, and extension at 72°C for 15 seconds, with a final extension at 72°C for five minutes and hold at 4°C. PCR products were confirmed by agarose gel electrophoresis using a 100 bp DNA ladder. The expected product sizes were 200 bp and 252 bp for exons 3 and 6, respectively.

DNA sequencing (capillary-based)

The amplified PCR products were subjected to capillary-based Big-Dye terminator sequencing. Prior to sequencing, the products underwent cycle sequencing and plate processing.

Statistical analysis was performed using SPSS version 26 (IBM Corp., Armonk, New York). Data were compiled in Microsoft Excel (Microsoft Corporation, Redmond, Washington) and analyzed using descriptive statistics, including counts, percentages, means, and standard deviations. Independent sample t-tests were used to compare normally distributed continuous variables between groups. 

Data were compiled using Microsoft Excel and analyzed using SPSS Statistics version 26. Descriptive statistics, including frequencies, percentages, means, and standard deviations, were used to summarize both categorical and continuous variables. The chi-square (χ²) test was applied to assess associations between categorical variables, including age groups, sex, alcohol consumption duration, disease stage, and genetic mutation status. For comparison of continuous variables between participants with and without genetic mutations, independent sample t-tests were conducted for parameters, such as BMI, liver function tests (AST, ALT, alkaline phosphatase (ALP), bilirubin, albumin, and globulin), hematological indices (hemoglobin, total leukocyte count, and platelet count), and other biochemical markers. A p-value of less than 0.05 was considered statistically significant.

## Results

The study included 60 patients diagnosed with alcohol-related liver disease. The majority of participants were aged 41-50 years (25 (41.7%)), followed by 31-40 years (19(31.7%)), 51-60 years (9(15.0%)), and 20-30 years (7(11.7%)). Most participants were male (58(96.7%)), with only 2(3.3%) being female. Regarding alcohol consumption history, 35(58.3%) had been consuming alcohol for 11-20 years, 19(31.7%) for less than 10 years, and 6(10.0%) for more than 20 years (Table 2).

**Table 2:** Baseline characteristics of study participants (n = 60). Frequency and percentages were calculated for all variables.

Characteristic	Category	Frequency (n)	Percentage (%)
Age (years)	20–30	7	11.7
	31–40	19	31.7
	41–50	25	41.7
	51–60	9	15.0
Sex	Male	58	96.7
	Female	2	3.3
Years of alcohol consumption	<10 years	19	31.7
	11–20 years	35	58.3
	>20 years	6	10.0

With respect to disease staging, cirrhosis was the most common diagnosis and was observed in 42(70.0%) participants. This was followed by steatohepatitis in 16(26.7%), while alcoholic gastritis and steatosis were each reported in 1(1.7%) participant (Table 3).

**Table 3:** Distribution of study participants by disease stage (n = 60). Frequency and percentages were calculated for all variables.

Disease stage	Frequency (n)	Percentage (%)
Alcoholic gastritis	1	1.7
Steatosis	1	1.7
Steatohepatitis	16	26.7
Cirrhosis	42	70.0

Genetic analysis showed that 49(81.7%) participants had no detectable mutations. The rs738409 polymorphism was found in 5(8.3%) individuals, while both rs738409 and rs738408 mutations were present in 6(10.0%) of the study participants (Table 4).

**Table 4:** Genetic polymorphism distribution among participants (n = 60). Frequency and percentages were calculated for all variables.

Genetic mutation	Frequency (n)	Percentage (%)
No mutation detected	49	81.7
rs738409	5	8.3
rs738409 + rs738408	6	10.0

When evaluating associations between genetic mutation status and demographic or clinical variables, no significant differences were found. Among participants aged 20-30 years, 5(10.2%) had no mutations, while 2(18.2%) had mutations. In the 31-40 year group, 15(30.6%) were mutation-negative, and 4(36.4%) were mutation-positive. In the 41-50 age range, 22(44.9%) had no mutations and 3(27.3%) had mutations. Lastly, in the 51-60 age group, 7(14.3%) were mutation-negative and 2(18.2%) had mutations (χ² = 1.34, p = 0.719). Among females, 2(4.1%) were mutation-negative and none were mutation-positive, while among males, 47(95.9%) were negative and 11(100.0%) were mutation-positive (χ² = 0.46, p = 0.496). Regarding alcohol use duration, 16(32.7%) mutation-negative and 3(27.3%) mutation-positive participants had consumed alcohol for less than 10 years; 28(57.1%) negative and 7(63.6%) positive had a history of 11-20 years; and 5(10.2%) negative and 1(9.1%) positive participants had consumed alcohol for over 20 years (χ² = 1.58, p = 0.924). Distribution by disease stage showed that among mutation-negative individuals, 1(2.0%) had alcoholic gastritis, 1(2.0%) had steatosis, 15(30.6%) had steatohepatitis, and 32(65.3%) had cirrhosis, while among those with mutations, none had gastritis or steatosis, 1(9.1%) had steatohepatitis, and 10(90.9%) had cirrhosis (χ² = 2.99, p = 0.702) (Table 5).

**Table 5:** Association between demographic and clinical variables and genetic mutation status. Chi-square test was applied, and a p-value of less than 0.05 was considered significant.

Variable	Category	No mutation (n = 49)	Mutation present (n = 11)	χ² value	P-value
Age (years)	20–30	5(10.2%)	2(18.2%)	1.34	0.719
	31–40	15(30.6%)	4(36.4%)		
	41–50	22(44.9%)	3(27.3%)		
	51–60	7(14.3%)	2(18.2%)		
Sex	Male	47(95.9%)	11(100%)	0.46	0.496
	Female	2(4.1%)	0(0%)		
Years of alcohol use	<10 years	16(32.7%)	3(27.3%)	1.58	0.924
	11–20 years	28(57.1%)	7(63.6%)		
	>20 years	5(10.2%)	1(9.1%)		
Disease stage	Gastritis	1(2.0%)	0(0%)	2.99	0.702
	Steatosis	1(2.0%)	0(0%)		
	Steatohepatitis	15(30.6%)	1(9.1%)		
	Cirrhosis	32(65.3%)	10(90.9%)		

Comparison of laboratory values between participants with and without genetic mutations revealed significantly higher AST levels in the mutation-positive group (213.09 ± 287.67 vs. 112.76 ± 106.28 U/L; t = 2.38, p = 0.020). Total leukocyte count was lower among mutation-positive individuals (8767.27 ± 2899.11 vs. 11,463 ± 7257/mm³; t = 2.52, p = 0.016), and platelet count was also significantly lower (94.73 ± 60.44 vs. 142.82 ± 85.53 ×10⁹/L; t = 3.23, p = 0.002). No statistically significant differences were observed in BMI, bilirubin levels, albumin, globulin, ALT, ALP, or hemoglobin between the two groups (Table 6).

**Table 6:** Comparison of laboratory parameters based on genetic mutation status. Mean and SD were calculated for all variables. T-test was applied, and a p-value of less than 0.05 was considered significant.

Laboratory parameter	Reference range	No mutation (n = 49)	Mutation present (n = 11)	t-value	P-value
BMI (kg/m²)	18.5–24.9	22.06 ± 3.30	21.74 ± 3.37	0.48	0.630
Total bilirubin (mg/dL)	0.2–1.2	7.07 ± 8.60	7.93 ± 8.51	0.51	0.616
Conjugated bilirubin (mg/dL)	0.0–0.3	5.19 ± 7.80	5.85 ± 7.77	0.43	0.672
Unconjugated bilirubin (mg/dL)	0.1–1.0	1.89 ± 1.65	1.97 ± 1.12	0.29	0.777
Albumin (g/dL)	3.4–5.4	2.68 ± 0.80	2.76 ± 0.86	0.48	0.630
Globulin (g/dL)	2.0–3.5	3.42 ± 0.78	3.65 ± 1.00	1.29	0.202
AST (U/L)	10–40	112.76 ± 106.28	213.09 ± 287.67	2.38	0.020
ALT (U/L)	7–56	80.67 ± 246.19	63.64 ± 56.96	0.47	0.634
ALP (U/L)	44–147	151.96 ± 71.03	138.73 ± 51.58	1.09	0.280
Hemoglobin (g/dL)	12–17.5	9.23 ± 2.80	9.50 ± 3.02	0.47	0.644
Total leukocyte count (/mm³)	4000–11,000	11,463 ± 7257	8767 ± 2899	2.52	0.016
Platelet count (×10⁹/L)	150–450	142.82 ± 85.53	94.73 ± 60.44	3.23	0.002

## Discussion

In the present study, the majority of participants with alcohol-related liver disease (ALD) were between 41 and 50 years of age (25 (41.7%)), followed by those aged 31-40 years (19(31.7%)). The mean age was 42.58 ± 8.7 years. A marked male predominance was observed, with 58(96.7%) participants being male and only 2(3.3%) being female. This is consistent with previous findings by Liangpunsakul et al., who highlighted a significant male dominance in alcoholic hepatitis (AH), likely due to higher levels of alcohol consumption among men [11].

Regarding alcohol consumption history, 35 participants (58.3%) had been consuming alcohol for 11-20 years, while 19(31.7%) had consumed alcohol for less than 10 years. Although alcohol exposure duration is a known risk factor, Liangpunsakul et al. also noted that beyond a certain threshold, neither the duration nor the quantity of alcohol consumed directly correlates with the severity of ALD. Instead, genetic predispositions and individual susceptibility appear to play a more critical role in disease progression [11].

Disease staging revealed that 42 patients (70.0%) had progressed to cirrhosis, while 16(26.7%) had steatohepatitis. These results are comparable to those reported by Malviya et al., who found cirrhosis in 70% of ALD cases in a large tertiary care center in India, with 28% of them in a decompensated state. Another Indian study reported that approximately 38.3% of chronic heavy drinkers eventually developed liver cirrhosis [12]. These findings reflect the late presentation of patients in the Indian healthcare setting and underscore the burden of advanced liver disease associated with chronic alcohol use.

Genetic analysis showed that five patients (8.3%) had the rs738409 polymorphism alone, and six patients (10.0%) carried both rs738409 and rs738408 variants. Overall, 18.3% of the cohort had the rs738409 variant. In a study by Zhang et al., involving 507 ALD patients and 645 healthy controls from the Han Chinese population, the rs738409 G allele was found to be significantly more common in ALD patients, with an odds ratio (OR) of 1.93, nearly doubling the risk of developing ALD [13]. Similarly, a European study involving 330 ALD patients and 328 controls reported that the rs738409 G allele was significantly associated with ALD (OR = 1.54) and with an increased risk of steatosis, fibrosis, and cirrhosis. Multivariate analysis identified the rs738409 variant as an independent predictor of cirrhosis risk (OR = 2.08) [13].

Further evidence from a U.S. study by Kolla et al. demonstrated that the rs738409 variant remained strongly associated with ALD among chronic drinkers, even after accounting for confounders like diabetes. This reinforces the pathogenic role of this single-nucleotide polymorphism in predisposing individuals to ALD [14].

This study has several limitations. First, the sample size was relatively small (n = 60), which may limit the generalizability of the findings. Second, the genetic analysis was confined to only two variants of the PNPLA3 gene (rs738409 and rs738408), potentially overlooking other relevant genetic polymorphisms involved in ALD pathogenesis. Third, the study did not explore the contribution of environmental or lifestyle-related factors, such as diet, tobacco use, or metabolic comorbidities, all of which may influence disease progression and severity independently of alcohol or genetic risk.

## Conclusions

This study demonstrates a significant association between PNPLA3 gene variants, particularly the rs738409 polymorphism, and the development of alcoholic liver disease (ALD), with a possible link to more advanced disease stages such as steatohepatitis and cirrhosis. These findings reinforce existing evidence on the pathogenic role of PNPLA3 in liver injury, fibrosis, and fat accumulation. Although a substantial proportion of patients with ALD carried the rs738409 variant, no statistically significant associations were observed between genetic mutation status and demographic variables, disease stage, or duration of alcohol consumption. This suggests that genetic predisposition may act independently of traditional risk factors, underscoring the need for broader genetic screening and personalized risk assessment in alcohol-related liver disease.
